# Stress Mindset and Psychological Distress in Primary and Secondary School Teachers: The Serial Mediating Role of Future Control and Burnout

**DOI:** 10.3390/healthcare14193137

**Published:** 2026-09-22

**Authors:** Xinwei Bei, Ning Liu, Yidan Yin, Yi Yang, Jinrun Wu, Qianfeng Li, Wan Zhao, Jia Wu

**Affiliations:** 1 School of Psychology, Hainan Normal University, Haikou 571158, China; 2 Adolescent Psychological Development and Education Center of Hainan, Hainan Normal University, Haikou 571158, China; 3 School of Economics and Finance, Xi’an International Studies University, Xi’an 710128, China; 4 School of Psychology, Nanjing Normal University, Nanjing 210097, China; 5 School of Elementary Education, Hainan Normal University, Haikou 571158, China

**Keywords:** stress-is-enhancing mindset, psychological distress, future control, burnout, primary and secondary school teachers

## Abstract

**Highlights:**

**What are the main findings?**

- A stress-is-enhancing mindset was associated with lower psychological distress among primary and secondary school teachers.
- Future control and burnout serve as independent and serial mediators in the relationship between the stress-is-enhancing mindset and psychological distress.

**What are the implications of the main findings?**

- Interventions aimed at cultivating a stress-is-enhancing mindset and future control, while reducing burnout, may represent a future direction for research on improving teachers’ mental health.
- The findings offer theoretical insights that may inform school-based mental health programs targeting teachers’ negative emotions.

**Abstract:**

**Background/Objectives**: As China prioritizes a high-quality teacher workforce, teachers’ mental health has gained growing attention. The stress-is-enhancing mindset has been linked to adaptive psychological functioning, but evidence among teachers remains limited. Drawing on conservation of resources theory, this study examined the associations among the stress-is-enhancing mindset, future control, burnout, and psychological distress, focusing on potential psychological processes related to teachers’ mental health. **Methods**: A sample of 1082 primary and secondary school teachers from four provinces in China completed measures of the stress-is-enhancing mindset, future control, burnout, and psychological distress. Indirect associations among these variables were examined. **Results**: The findings indicated that the stress-is-enhancing mindset was associated with lower psychological distress and burnout, as well as greater future control. The association between the stress-is-enhancing mindset and psychological distress involved indirect associations through future control and burnout, including a sequential indirect pathway. **Conclusions**: The stress-is-enhancing mindset is associated with lower psychological distress, with indirect associations observed through future control and burnout. Because the data were cross-sectional, the temporal ordering and causal status of these pathways remain to be established.

## 1. Introduction

Ongoing educational reform has intensified role demands and occupational stress among Chinese primary and secondary teachers, with documented adverse effects on mental health [1]. Psychological distress—the unique discomfort, emotional state experienced by an individual in response to a specific stressor or demand that results in harm, either temporary or permanent, to the person [2,3]—encompasses depression, anxiety, and stress symptoms [4,5]. Epidemiological data show depressive symptom prevalence of 18.3% in primary and 20.1% in secondary teachers, and anxiety symptom prevalence of 14.7% and 18.9%, respectively [6,7]. These figures suggest that teacher psychological distress is a notable occupational health concern. Given its consequences for teachers and students alike [8], understanding factors that support teacher well-being is critical for sustaining the teaching workforce. International research shows that teacher burnout is associated with poorer well-being and greater intentions to leave the profession, highlighting the need to identify psychological resources that may support teachers’ occupational sustainability [9]. Therefore, identifying psychological resources that support teacher well-being and occupational sustainability has become an important research priority.

Stress has long been seen as something harmful in both popular culture and academic research. But many important and meaningful life experiences are inevitably stressful. A stress mindset refers to individuals’ beliefs about whether stress has enhancing or debilitating effects. People who hold a stronger stress-is-enhancing mindset believe that stress can facilitate performance, health, well-being, and learning [10], and they tend to report better health, fewer depressive and anxious symptoms, and higher energy levels [11]. In contrast, those with a stronger stress-is-debilitating mindset usually experience more severe psychological distress when facing stressors [12]. Studies have shown that stress-is-enhancing is significantly correlated with mental health among adolescents [13], and that it may weaken the relationship between negative life events and depressive symptoms [14]. Therefore, it is possible that a stress-is-enhancing mindset protects mental health by reducing rumination and psychological distress [11]. Based on these findings, the present study aims to examine how the stress-is-enhancing mindset relates to mental health among primary and secondary school teachers, and to explore its potential pathways.

In occupational health psychology, the stress-is-enhancing mindset is regarded as an important cognitive resource that protects mental health under work stress. It buffers the negative effect of work–family conflict on hope, thereby reducing burnout risk [15], and may also protect teachers’ physical and mental health by alleviating the sense of time resource scarcity [16]. According to stress mindset theory, individuals holding a stress-is-enhancing mindset tend to perceive challenges as opportunities, and thus demonstrate stronger capacity for goal planning and resource allocation. However, while prior research has suggested that this mindset may be related to occupational health among primary and secondary school teachers, how it reduces psychological distress through resource-related mechanisms remains insufficiently examined, especially with few systematic studies using multiple indicators [17]. Based on previous research, the present study proposes a hypothesis.

**Hypothesis** **1.** *A stress-is-enhancing mindset is negatively associated with psychological distress among primary and secondary school teachers.*

A sense of control over the external environment and future events may be an important pathway linking the stress mindset and mental health. Future control reflects individuals’ subjective beliefs about their own developmental trajectory and future outcomes, and is viewed as a key resilience resource [18]. Individuals with a higher sense of future control believe they can actively shape their developmental direction, and tend to approach challenges with a positive and rational attitude when facing stress, deriving adaptation and growth from the experience. Theoretically, teachers’ stress-is-enhancing mindset may facilitate the formation of future control, which in turn plays an important role in maintaining mental health. Empirical studies have shown that a greater sense of control is associated with fewer mental health symptoms; enhancing subjective control can effectively buffer the negative effects of stress; and higher levels of control are linked to lower trait anxiety and depressive symptoms [19]. When individuals perceive the future as lacking options, vague, and negative, their worries about uncertainty increase, placing them at greater risk for depression, anxiety, and stress [20]. Longitudinal studies have also found that positive future perception and sense of control during adolescence significantly predict lower depressive symptoms in adulthood [21]. However, whether future control serves as an effective protective factor for mental health among primary and secondary school teachers has not been systematically examined. Thus, exploring the mechanism of future control in teachers’ mental health may help enrich relevant theories and provide a reference for practical interventions in teacher mental health. Based on the above, the present study proposes the following hypothesis.

**Hypothesis** **2.** *Future control mediates the relationship between the stress-is-enhancing mindset and psychological distress among primary and secondary school teachers.*

Burnout is an adverse reaction to chronic stress in the workplace, characterized by prolonged emotional and interpersonal strain. It can be summarized into three dimensions: exhaustion, cynicism, and reduced efficacy [22]. Globally, burnout not only poses potential risks to teachers’ health but also serves as a key risk factor for the deterioration of their physical and mental well-being [23]. When teachers experience burnout, they are less willing to invest time and energy in students, which may negatively affect student development [24].

First, the stress-is-enhancing mindset may be significantly negatively correlated with burnout. Burnout is a negative consequence of work stress, and a stress-is-enhancing mindset has been considered a potential resource for preventing burnout [25]. Empirical research has found that the stress-is-enhancing mindset can buffer the damaging effect of work–family conflict on hope among primary and secondary school teachers, thereby leading to lower levels of burnout [15]. According to conservation of resources theory, individuals strive to acquire, maintain, and protect valuable resources, and that resource loss or insufficient resource gain may contribute to psychological strain [26]. From this perspective, the stress-is-enhancing mindset may represent an adaptive psychological resource that enables individuals to perceive stress as an opportunity for growth rather than a threat, thereby facilitating the mobilization and accumulation of other personal resources. Proactive coping styles that directly face challenges have been linked to lower levels of burnout [27]. Previous research has shown that viewing stress as enhancing predicts lower levels of burnout among school teachers [28].

Furthermore, existing studies have demonstrated a significant association between burnout and psychological distress, including depression and anxiety symptoms [29,30]. Among teachers, higher levels of burnout have been consistently linked to poorer mental health outcomes and reduced psychological well-being. Recent systematic reviews have further highlighted teacher burnout as an important occupational health concern associated with adverse psychological outcomes and impaired well-being [8,23]. Based on the above, the present study proposes the following hypothesis.

**Hypothesis** **3.** *Burnout mediates the relationship between the stress-is-enhancing mindset and psychological distress among primary and secondary school teachers.*

Individual’s sense of control over the future may influence how they cope with stress and how well they preserve resources. Future control—the belief that one can manage future life and achieve goals—helps individuals maintain core resource reserves under stress and prevents resource depletion [31,32]. As a future-oriented form of perceived control, future control reflects individuals’ beliefs about their ability to influence future outcomes and may be associated with teacher burnout. Individuals with higher future control tend to adopt more proactive coping strategies when facing stress, thereby reducing burnout and psychological distress [33]. Conversely, lack of future orientation may weaken long-term goal pursuit and increase burnout, whereas individuals with future-oriented traits are better at regulating negative emotions and reducing burnout risk through goal planning and implementation [34]. From the perspective of conservation of resources theory, the stress-is-enhancing mindset may function as a psychological resource that shapes individuals’ interpretations of stressful experiences. Teachers who view stress as potentially beneficial may be more likely to perceive greater control over their future and maintain psychological resources when facing occupational challenges.

Drawing on conservation of resources theory, the stress-is-enhancing mindset may not only influence psychological distress directly through burnout, but also operate through a sequential pathway—enhancing future control, which in turn reduces burnout, and ultimately alleviates psychological distress. Research has shown that work control can protect individuals from adverse work environments and buffer the psychological strain of high job demands; the stronger the perceived autonomy, the more effectively work control reduces emotional exhaustion and depersonalization [35]. Thus, teachers’ perceived control over their future work outcomes may be a key psychological resource that alleviates burnout—the stronger the sense of future control, the lower the level of burnout. Based on these considerations, the present study proposes the following hypothesis.

**Hypothesis** **4.** *The stress-is-enhancing mindset is associated with psychological distress among primary and secondary school teachers through the serial mediation of future control and burnout.*

The present study aims to examine the potential psychological processes underlying the association between the stress-is-enhancing mindset and teachers’ psychological distress, and to provide theoretical support and practical implications for improving teachers’ mental health and enhancing school-based mental health intervention systems. The hypothesized serial mediation model is presented in Figure 1. In addition, age and gender were included as covariates because previous research has reported demographic differences in teachers’ psychological distress, stress-related experiences, and burnout [36].

## 2. Materials and Methods

### 2.1. Participants and Procedure

In the current survey, a total of 1082 primary and secondary school teachers were recruited from Henan Province, Jiangsu Province, Chongqing Municipality, and Beijing, China. The selection of participating counties/districts and schools was based on regional diversity and accessibility through established research collaborations. The sample included 291 male teachers (26.9%) and 791 female teachers (73.1%), with ages ranging from 22 to 60 years (M = 37.18, SD = 8.56). Participants reported an average teaching experience of 14.73 years (SD = 10.27; *n* = 1079 due to missing responses). Among them, 424 teachers (39.2%) worked in primary schools and 658 (60.8%) worked in secondary schools. Regarding school location, 648 teachers (59.9%) were from rural schools and 434 (40.1%) were from urban schools. Participants were recruited from Beijing and Jiangsu Province in the eastern region (*n* = 496, 45.8%), Henan Province in the central region (*n* = 345, 31.9%), and Chongqing Municipality in the western region (*n* = 241, 22.3%). All participants provided written informed consent before completing the questionnaire.

A multi-stage sampling strategy was adopted to recruit participants from primary and secondary schools. First, four provincial-level regions were selected based on China’s national classification of economic regions, including Beijing and Jiangsu Province representing eastern China, Henan Province representing central China, and Chongqing representing western China. Subsequently, one county or district within each selected region was chosen for further investigation. Within each selected county or district, 51 primary and secondary schools were recruited through local educational contacts. In schools with a large teaching staff (over 60 teachers), approximately 50–70% of teachers were randomly selected. In medium-sized schools (with 30–60 teachers), stratified sampling was applied based on available teacher characteristics. For small schools (fewer than 30 teachers), all available teachers were invited to participate. Consistent with ethical requirements, participation was voluntary, and only teachers who provided informed consent completed the questionnaire. Participants completed an electronic questionnaire that required approximately 15 min. Upon completion, participants received 20 RMB as compensation for their participation. This study was conducted in accordance with the ethical guidelines for research involving human participants and was approved by the Ethics Committee of Hainan Normal University.

### 2.2. Measurement

#### 2.2.1. Stress-Is-Enhancing Mindset

The stress-is-enhancing mindset was measured using an established Chinese version of the scale developed by Crum et al. (2013) [11], which has demonstrated satisfactory psychometric properties in previous studies. This scale is unidimensional and consists of eight items. Sample items include: “The effects of stress are negative and should be avoided,” “Experiencing stress facilitates my learning and growth,” and “Experiencing stress impairs my health and vitality.” Items were rated on a 5-point Likert scale ranging from 0 (strongly disagree) to 4 (strongly agree), with 2 representing neither agree nor disagree. Items 1, 3, 5, and 7 were reverse-scored. Higher total scores indicate higher levels of stress-is-enhancing mindset. In the present study, the Cronbach’s α coefficient for this scale was 0.67.

#### 2.2.2. Future Control

Future control was assessed using the Chinese version of the Perceived Control over the Future Scale [18]. The wording of the items was adapted to make them suitable for primary and secondary school teachers while retaining the original meaning. The adapted items were reviewed by experts, and previous studies have supported the reliability and validity of this measure. A sample item is “I am confident about my future; my future is largely in my own hands.” The scale consists of seven items, each rated on a 4-point scale ranging from 1 (strongly disagree) to 4 (strongly agree). Higher average scores indicate higher levels of future control. In the present study, the Cronbach’s α coefficient for this scale was 0.79.

#### 2.2.3. Burnout

Burnout was measured using the Maslach Burnout Inventory–Educators Survey (MBI-ES), originally developed by Maslach and Jackson [22], and the Chinese version of the MBI has demonstrated satisfactory reliability and validity among Chinese teachers [37]. The MBI consists of 22 items assessing emotional exhaustion, depersonalization, and reduced personal accomplishment [22]. Consistent with the multidimensional conceptualization of burnout, the three dimensions were combined to represent teachers’ overall burnout [38,39]. Sample items include: “I feel emotionally drained from my work,” “I don’t really care what happens to some students,” and “I deal very effectively with the problems of my students.” Participants responded based on their actual feelings at work using a 7-point scale ranging from 0 (never) to 6 (every day). The personal accomplishment items were reverse-scored so that higher scores consistently indicated higher levels of burnout. The three dimensions were then averaged to obtain an overall burnout score. In the present study, the Cronbach’s α coefficient for this scale was 0.91. The emotional exhaustion, depersonalization, and reduced personal accomplishment subscales demonstrated good internal consistency in the present study (Cronbach’s α = 0.90, 0.90, and 0.88, respectively).

#### 2.2.4. Psychological Distress

Psychological distress was measured using the Chinese version of the Depression Anxiety Stress Scales-21 [40]. The scale consists of three subscales—depression, anxiety, and stress—each containing seven items, for a total of 21 items. Items are rated on a 4-point Likert scale ranging from 0 (does not apply to me) to 3 (applies to me very much), with 1 indicating applies to me to some degree and 2 indicating applies to me to a considerable degree. Psychological distress was calculated as the mean score of the 21 DASS items, with higher mean scores indicating greater psychological distress. In the present study, the Cronbach’s α coefficient for this scale was 0.97.

### 2.3. Data Analysis Process

Since all data in this study were collected through self-report measures, common method bias may have been a concern. To minimize its potential influence, we implemented several procedural controls prior to the survey, including anonymous administration and the inclusion of reverse-coded items. After data collection, Harman’s single-factor test was conducted as a preliminary assessment of common method variance. Eight factors with eigenvalues greater than 1 were extracted without rotation, and the first factor accounted for 33.68% of the total variance. Although the first factor did not account for the majority of variance, this test cannot completely rule out the possibility of common method variance.

Before data analysis, the data were screened for missing values and invalid responses. A small number of missing responses were observed for some demographic variables, and available valid responses were used. No cases were excluded due to missing data on the main study variables. Potential outliers and regression assumptions were also examined before the main analyses. The demographic description, correlation analysis, and mediation model analysis were conducted using SPSS 26.0 and the PROCESS macro for SPSS [41]. Pearson correlation analyses were conducted using continuous composite scores of all variables. A confirmatory factor analysis was conducted using Mplus 7. Pearson correlation analysis was used to examine the relationships among the main variables. A confirmatory factor analysis was conducted to examine the distinctiveness of the four study constructs. Given that teachers were nested within schools, intraclass correlations (ICCs) were calculated to evaluate the extent of school-level clustering. In addition, a sensitivity analysis was conducted using Mplus with school-level cluster-robust standard errors (TYPE = COMPLEX, MLR estimator) to examine whether accounting for the nested data structure influenced the mediation results. The chain mediation effect was tested using Model 6 of the PROCESS v4.1 macro. To test the significance of the indirect effects, we employed a bias-corrected percentile bootstrap method with 5000 resamples to construct 95% confidence intervals (CIs). An indirect effect was considered statistically significant if its 95% CI did not contain zero. Age and gender were included as demographic covariates due to their potential relevance to teachers’ psychological well-being; however, covariate adjustment does not eliminate all potential bias.

## 3. Results

### 3.1. Descriptive Statistics and Correlation Analysis

Before conducting the main analyses, data screening was performed to assess distributional assumptions. Skewness and kurtosis values were examined for all continuous variables. The results indicated acceptable distributions, with skewness values ranging from −0.087 to 0.837 and kurtosis values ranging from −1.064 to 2.971. Moreover, the means (M), standard deviations (SDs), and Pearson correlation coefficients for all variables are presented in Table 1. The results showed that teachers’ stress-is-enhancing mindset was significantly negatively correlated with psychological distress (*r* = −0.30, *p* < 0.001) and burnout (*r* = −0.41, *p* < 0.001), and significantly positively correlated with future control (*r* = 0.36, *p* < 0.001). Burnout was significantly positively correlated with psychological distress (*r* = 0.63, *p* < 0.001), and future control was also significantly negatively correlated with psychological distress (*r* = −0.50, *p* < 0.001). In addition, burnout was significantly negatively correlated with future control (*r* = −0.52, *p* < 0.001). These findings provided preliminary support for the subsequent mediation analyses. Gender and age were entered as control variables in the subsequent regression analyses.

### 3.2. Mediation Model Analysis

To further examine the discriminant validity of the four focal constructs, we conducted confirmatory factor analyses comparing the hypothesized four-factor model with alternative three-factor and one-factor models. The results of the confirmatory factor analysis indicated that the hypothesized four-factor model demonstrated a better fit to the data than alternative models combining burnout and psychological distress or treating all constructs as a single factor. Specifically, the four-factor model showed a substantially better fit (χ^2^ = 588.747, df = 48, CFI = 0.928, TLI = 0.901, SRMR = 0.068) than the three-factor model (χ^2^ = 1183.666, df = 51, CFI = 0.849, TLI = 0.804, SRMR = 0.083) and the one-factor model (χ^2^ = 2788.577, df = 54, CFI = 0.634, TLI = 0.553, SRMR = 0.141), supporting the discriminant validity of the four constructs. Moreover, because teachers were nested within schools, we examined the extent of school-level clustering. The ICCs for the stress-is-enhancing mindset, future control, burnout, and psychological distress were 0.005, 0.022, 0.040, and 0.072, respectively, indicating limited between-school variance.

To further explore the underlying mechanism through which the stress-is-enhancing mindset affects primary and secondary school teachers’ psychological distress, this study constructed a chain mediation model with the stress-is-enhancing mindset as the independent variable, psychological distress as the dependent variable, and future control and burnout as serial mediators. Gender and age were included as control variables. The overall regression model was significant, *R*^2^ = 0.10, *F* (3, 1078) = 37.86, *p* < 0.001. The total effect of the stress-is-enhancing mindset on psychological distress was significant (β = −0.39, t = −10.45, *p* < 0.001). After entering future control and burnout as mediators, the direct effect of the stress-is-enhancing mindset on psychological distress was not significant (β = −0.01, t = −0.30, *p* = 0.76).

The standardized path coefficients are presented in Figure 2, whereas unstandardized regression coefficients (B) are reported in the text. The stress-is-enhancing mindset was positively associated with future control (B = 0.33, SE = 0.03, t = 12.68, *p* < 0.001, 95% CI = [0.277, 0.379]). Future control was negatively associated with burnout (B = −1.01, SE = 0.06, t = −16.32, *p* < 0.001, 95% CI = [−1.128, −0.886]), and burnout was positively associated with psychological distress (B = 0.32, SE = 0.02, t = 18.88, *p* < 0.001, 95% CI = [0.283, 0.349]). The stress-is-enhancing mindset was also negatively associated with burnout (B = −0.54, SE = 0.06, t = −9.67, *p* < 0.001, 95% CI = [−0.653, −0.433]), whereas future control was negatively associated with psychological distress (B = −0.31, SE = 0.04, t = −8.06, *p* < 0.001, 95% CI = [−0.379, −0.231]). However, the stress-is-enhancing mindset was not significantly associated with psychological distress after accounting for future control and burnout (B = −0.01, SE = 0.03, t = −0.30, *p* = 0.766, 95% CI = [−0.073, 0.054]). The regression models and corresponding *R*^2^ values are presented in Table 2.

To further examine the indirect associations among these variables, bootstrap analyses were conducted for the three specific and sequential indirect pathways. The bootstrap results for the three indirect paths are summarized in Table 3. First, the stress-is-enhancing mindset showed a negative indirect association with psychological distress through future control (indirect effect = −0.100, 95% CI = [−0.137, −0.068]). Second, the stress-is-enhancing mindset showed a negative indirect association with psychological distress through burnout (indirect effect = −0.172, 95% CI = [−0.217, −0.131]). Third, the stress-is-enhancing mindset showed a negative sequential indirect association with psychological distress through future control and burnout (indirect effect = −0.104, 95% CI = [−0.132, −0.080]). However, the direct association between the stress-is enhancing mindset and psychological distress was not significant (the direct effect was −0.010, 95% CI = [−0.0727, 0.0535]).

In addition, sensitivity analyses using school-level cluster-robust standard errors yielded results consistent with the original mediation model. Specifically, the stress-is-enhancing mindset was positively associated with future control (β = 0.359, 95% CI [0.287, 0.432]) and negatively associated with burnout (β = −0.255, 95% CI [−0.306, −0.205]). Future control was negatively associated with burnout (β = −0.432, 95% CI [−0.474, −0.390]) and psychological distress (β = −0.219, 95% CI [−0.302, −0.136]), whereas burnout was positively associated with psychological distress (β = 0.529, 95% CI [0.457, 0.601]). Taken together, these findings suggest that the stress-is-enhancing mindset was indirectly associated with primary and secondary school teachers’ psychological distress through future control and burnout, including specific indirect associations and a sequential indirect association through the two mediators, whereas its direct association with psychological distress was not significant.

## 4. Discussion

This study examined how the stress-is-enhancing mindset relates to psychological distress among primary and secondary teachers. The results showed that the stress-is-enhancing mindset was indirectly associated with psychological distress through future control and burnout, including both specific indirect associations and a sequential indirect pathway involving these variables. These findings extend stress mindset theory by highlighting the potential psychological processes through which a stress mindset may relate to teachers’ mental health in demanding work contexts. Specifically, a positive stress mindset may be associated with greater future control and reduced negative work-related emotional states. By focusing on teachers—a chronically stressed population—this study fills a gap in the literature, which has relied heavily on college student samples [12]. The results also provide clear implications for designing targeted mental health interventions for teachers.

The present study found that the stress-is-enhancing mindset was negatively associated with psychological distress, which is consistent with previous findings [42]. Teachers who hold this mindset tend to interpret work stress as a manageable challenge that facilitates growth, which may contribute to better psychological adjustment [15]. Viewing stress as a challenge rather than a threat, and believing that overcoming obstacles leads to gains, appears to promote psychological well-being [43]. Indeed, individuals can achieve enhanced functioning after traumatic experiences [44]. For teachers facing high work stress, this cognitive shift is particularly important. It encourages proactive coping rather than passive endurance. Overall, these findings suggest that the stress mindset is an important factor associated with teachers’ mental health, while the psychological processes underlying this association warrant further investigation.

Beyond the overall association between the stress-is-enhancing mindset and psychological distress, the present study further examined the potential role of future control in this association. Specifically, the stress-is-enhancing mindset was positively associated with future control, and future control was negatively associated with psychological distress. This finding suggests that a positive stress mindset strengthens individuals’ belief in their capacity to shape what lies ahead. Prior research indicates that such a mindset sustains hope under stress [15], and may reinforce teachers’ conviction that their actions and efforts can influence future outcomes [12]. This sense of control matters because it buffers against psychological distress. According to Beck’s cognitive theory, perceiving the future as constrained is central to depression [45]. Subjective future control has been shown to mediate the relationship between the stress mindset and mental health outcomes [19]. Conversely, when individuals view the future as uncertain or pessimistic, they become more vulnerable to depression and anxiety [20,21]. For teachers, believing that they can shape their own future and seeing it as full of opportunities rather than threats reduces worry about the unpredictable, thereby alleviating negative emotions. Thus, the stress-is-enhancing mindset, by strengthening future control, helps teachers reframe occupational challenges from a longer-term perspective and effectively is associated with lower psychological distress.

The present study found that the stress-is-enhancing mindset influenced psychological distress among primary and secondary school teachers through the mediating role of burnout, a result consistent with previous findings [46]. Teachers who hold a stress-is-enhancing mindset are more willing to actively engage with challenges at work, and are more likely to experience flow and engage in critical reflective practices, thereby reporting lower levels of burnout [15]. Although high expectations can sometimes lead to overinvestment and eventual exhaustion [27], a positive stress mindset appears to change how teachers perceive demanding situations, preventing cumulative strain from developing into burnout [44]. When teachers view stress as an opportunity for growth rather than a drain on personal resources, they are more likely to adopt mature coping strategies. This adaptive cognitive pattern may help teachers reinterpret stressful experiences more positively and is associated with lower levels of burnout [47]. In this regard, cultivating a stress-is-enhancing mindset may help protect teachers from burnout [15], and alleviate its emotional and physical consequences [23]. Even under high workloads, this mindset enables teachers to find meaning in stress and channel it toward personal growth, thereby reducing exhaustion. When resources are limited, it encourages them to actively seek or create alternatives rather than withdraw from their work [46]. Reduced burnout, in turn, is associated with lower levels of depression, anxiety, and stress.

Importantly, future control and burnout sequentially mediated the relationship between the stress-is-enhancing mindset and psychological distress, a pattern consistent with earlier work on future time perspective and burnout [16,32,34,48]. As a cognitive resource, the stress-is-enhancing mindset may enable teachers to interpret stressful experiences as potential opportunities for growth, which may contribute to resource preservation from the perspective of conservation of resources theory [46]. Successful coping then generates additional resources through a resource gain spiral. Teachers with adequate resource reservoirs are less vulnerable to loss, consistent with the resource loss priority principle. By strengthening future control, this mindset enables teachers to anticipate risks and allocate resources in advance, thereby interrupting negative emotional spirals. Overall, this serial mediation pathway illustrates the progressive process through which the stress-is-enhancing mindset is associated with better mental health among teachers and extends the application of COR theory to this population.

Several limitations should be noted. First, the cross-sectional design limits the ability to determine the temporal ordering and causal relationships among the stress-is-enhancing mindset, future control, burnout, and psychological distress. Although the proposed model was theoretically derived, longitudinal and experimental studies are needed to further examine these relationships. In addition, self-reported measures may still be subject to social desirability bias, and future studies should consider incorporating social desirability measures or multi-source assessments. Second, other individual and contextual factors may contribute to teachers’ psychological distress beyond the variables examined in this study, and future research should test the model across diverse cultural contexts and populations. Third, the internal consistency of the stress-is-enhancing mindset scale was relatively modest. Future studies should further examine the reliability and validity of this measure in diverse samples. Fourth, the cross-sectional design prevents us from determining the stability and malleability of the stress-is-enhancing mindset. Future research should examine whether the stress-is-enhancing mindset represents a stable belief system or a malleable framework that can be modified through targeted interventions to promote teachers’ psychological well-being.

Despite these limitations, the present study contributes to the literature in several ways. It highlights the potential roles of future control and burnout in explaining the association between the stress-is-enhancing mindset and psychological distress, thereby extending understanding of the psychological processes underlying teachers’ mental health. It also extends stress mindset research by focusing on primary and secondary school teachers, an occupational group whose mental health has received increasing attention but remains underexplored. These findings provide theoretical insights into how psychological resources and occupational experiences may be associated with teachers’ mental health, while future longitudinal and intervention studies are needed to evaluate causal pathways and practical applications.

## 5. Conclusions

In conclusion, this study provides evidence that the stress-is-enhancing mindset is associated with teachers’ psychological distress through potential indirect pathways involving future control and burnout. The findings highlight the potential role of adaptive stress appraisal and perceived future control in understanding teachers’ occupational well-being. However, given the cross-sectional design, the observed associations should not be interpreted as causal relationships. Future longitudinal and intervention studies are needed to further examine these relationships.

## Figures and Tables

**Figure 1:**
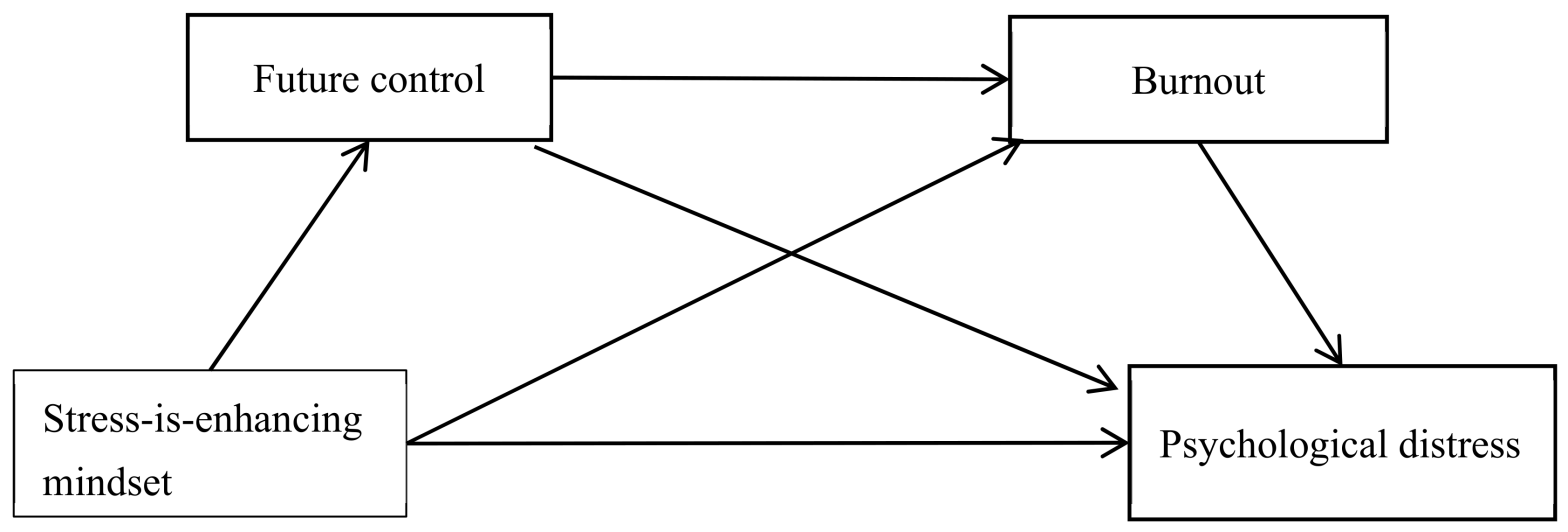
Hypothesized serial mediation model.

**Figure 2:**
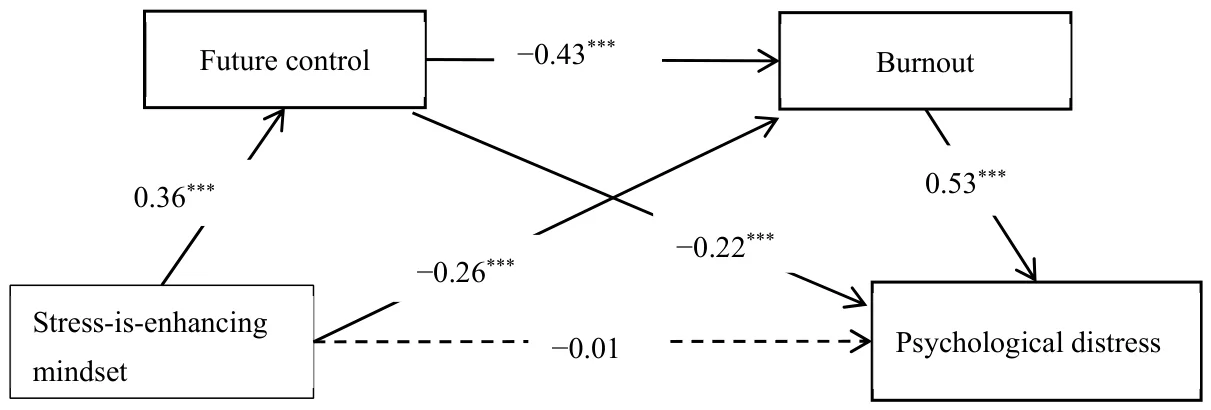
Serial mediation model. Note: *** *p* < 0.001.

**Table 1:** Descriptive statistics and correlation results for each variable.

Variables	1	2	3	4	5	6
1. gender	-					
2. age	−0.14 ***	-				
3. stress-is-enhancing mindset	−0.05	−0.01	-			
4. future control	0.07 *	−0.05	0.36 ***	-		
5. burnout	−0.07 *	−0.11 ***	−0.41 ***	−0.52 ***	-	
6. psychological distress	−0.03	0.05	−0.30 ***	−0.50 ***	0.63 ***	-
M	0.73	37.18	3.11	2.85	3.19	1.02
SD	0.44	8.56	0.48	0.44	1.02	0.61

Note: * *p* < 0.05, *** *p* < 0.001. Gender: 0 = male, 1 = female; age: 22–60 years. Scores were calculated as mean item scores, with higher scores indicating higher levels of the corresponding constructs.

**Table 2:** Regression models predicting future control, burnout, and psychological distress.

Predictor	Outcome		
	Future Control	Burnout	Psychological Distress
Constant	1.856	8.506	0.622
Gender	0.081	−0.156	0.034
Age	−0.002	−0.017	0.007
Stress-is-enhancing mindset	0.330	−0.540	−0.010
Future control	-	−1.010	−0.310
Burnout	-	-	0.320
*R* ^2^	0.136	0.349	0.450
*F*	56.512 ***	144.143 ***	176.286 ***
*df*	3, 1078	4, 1077	5, 1076

Note: *** *p* < 0.001. Values are unstandardized regression coefficients (B). Gender and age were included as covariates. *R*^2^ represents the proportion of variance explained by each regression model.

**Table 3:** Indirect associations of the stress-is-enhancing mindset with psychological distress through future control and burnout.

Effect Type	Intermediary Paths	Bootstrap 95% Confidence Interval	Effect Estimates
Direct Effect	stress-is-enhancing mindset → psychological distress	[−0.073, 0.054]	−0.010
	stress-is-enhancing mindset → future control → psychological distress	[−0.137, −0.068]	−0.100
Mediating Effect	stress-is-enhancing mindset → burnout → psychological distress	[−0.217, −0.131]	−0.172
	stress-is-enhancing mindset → future control → burnout → psychological distress	[−0.132, −0.080]	−0.104
Overall Mediating Effect		[−0.436, −0.321]	−0.376
Overall Effect		[−0.458, −0.313]	−0.386

## Data Availability

The data presented in this study are available on request from the corresponding author. The data are not publicly available due to privacy or ethical restrictions.
